# Intra- and intersession reliability and agreement of the Unilateral Seated Shot-Put Test outcome measures in healthy male athletes

**DOI:** 10.1186/s13102-021-00301-4

**Published:** 2021-07-06

**Authors:** Matthieu Degot, Yoann Blache, Grégory Vigne, Gabriel Franger, Lionel Neyton, Isabelle Rogowski

**Affiliations:** 1 Laboratoire Interuniversitaire de Biologie de la Motricité EA 7424, Université de Lyon, UFRSTAPS, 27-29 Boulevard du 11 Novembre 1918, 69622 Villeurbanne Cedex, France; 2 Athletic France, 4 rue Jean Sarrazin, 69008 Lyon, France; 3 Centre Orthopédique Santy, Fifa Medical Center of Excellence, 24 Avenue Paul Santy, 69008 Lyon, France; 4 Hôpital Privé Jean Mermoz, Ramsay-Générale de Santé, 55 avenue Jean Mermoz, 69008 Lyon, France

**Keywords:** Physical performance test, Upper limb power, Limb symmetry index, Minimum detectable change, Standard error of measurement

## Abstract

**Background:**

The Unilateral Seated Shot-Put Test (USSPT) consists of pushing an overweight ball as far as possible to assess upper extremity power unilaterally and bilateral symmetry. Literature however reports various body positions and upper limb pushing patterns to perform USSPT, demanding to provide additional guideline to achieve overweight ball push. This study therefore aimed at assessing the reliability and agreement of USSPT outcome measures when pushing an overweight ball in a horizontal direction.

**Methods:**

Twenty-seven healthy male athletes performed two sessions, one week apart, of three unilateral pushes per upper limb using a 3-kg medicine ball, for which the distances were measured. The intraclass correlation coefficient (ICC), standard error of measurement (SEM), minimum detectable change at a 95 % confidence level (MDC_95 %_) and coefficient of variation (CV) were assessed for the pushing distances based on one, two or three trials per side to produce two outcome measures: the pushing distance per limb and USSPT Limb Symmetry Index (LSI) when dividing pushing distance of the dominant side by that of the non-dominant side.

**Results:**

The most reliable pushing distance per limb was obtained when averaging three pushing distances, normalized by body mass with the exponent 0.35. The mean USSPT LSI was 1.09 ± 0.10 for the first session and 1.08 ± 0.10 for the second session, highlighting good reliability and agreement (ICC = 0.82; SEM = 0.045; MDC_95 %_ = 0.124; CV = 5.02 %).

**Conclusions:**

When the overweight ball is pushed in a horizontal direction, averaging the distances of three trials for both the dominant and non-dominant limbs is advised to provide the most reliable USSPT distance per limb and USSPT LSI.

## Background

Shoulder pain and injuries are frequently observed in overhead or contact sports. Prevalence rates of 40–91 % are reported in competitive swimmers [1] and 9–17 % of injuries are located at the shoulder complex in elite rugby players [2]. Conservative or surgical management of such injuries often results in time-loss in sport participation [3, 4]. After such a period, the return to sport at the preinjury level, while limiting the risk of recurrence, remains challenging for competitive athletes [5]. Implementing a battery of physical performance tests to assess shoulder functions in athlete’s follow-up may help coaches and clinicians in the preseason screening and for the return-to-sport decision-making [6]. However, additional knowledge on upper extremity functional tests still needs, particularly on the reliability of their outcome measures when they are implemented in easy-to-use conditions.

The power function of upper extremities is commonly assessed through the Unilateral Seated Shot-Put Test (USSPT), which consists of a forward pushing of an overweight ball in a seated position [7–9]. Previous studies however reported various seated positions to perform USSPT. Indeed, in Negrete et al. [7], participant sat on a chair (18-inch), while, in Chmielewski et al. [8] and Riemann et al. [9], participant sat on the floor with either knees flexed at right angles [8] or extended [9]. Another discrepancy concerns the trunk, which can be stabilized either using a strap around the chest [7], or with halfback support [8, 10] or with complete backrest [9]. Such variabilities in positioning may present some drawbacks, such as the time consumed to strap the participant, the extension of the medicine ball’s trajectory when sitting on a chair, or the limitation in the arm and scapular contributions when using a complete backrest. It then appears that the participant’s position when sitting on the floor with halfback support and knees bent at right angles with feet flat on the ground, as described by Chmielewski et al. [8], may be the easiest-to-use positioning while limiting drawbacks to implement USSPT in battery of upper extremity physical performance tests.

USSPT provides a pushing distance per side, which is independent of body mass when scaled allometrically [8]. This pushing distance presents high reliability in healthy [11] and symptomatic athletes [10], when the distances of three trials are averaged [8, 11, 12]. Performing three trials per side can nevertheless be time-consuming, especially when USSPT is included in a battery of tests, demanding to explore the reliability of the USSPT pushing distance per side when the distances of less trials are considered. In addition, the bilateral symmetry in the upper limb performance is usually quantified through the limb symmetry index (LSI) [8, 9]; but the LSI reliability and agreement have not been assessed yet for USSPT. Such an index may be however influenced by the differences in pushing patterns between sides, since a difference up to 8° in overweight-ball release angle has been observed between the dominant and non-dominant sides [9]. Providing an additional guideline, such as pushing in a horizontal direction, may help to reduce side-to-side differences when performing USSPT. Consequently, any changes in pushing motion achievement or in method to compute USSPT pushing distances per side, and then USSPT LSI, demand to explore the reliability and agreement of USSPT outcome measures before their use by coaches.

This study aimed at assessing the reliability and agreement of USSPT outcome measures based on one, two or three trials per side when pushing the overweight ball in a horizontal direction. It was hypothesized that this procedure meets reliability and agreement criteria when mean distances of two trials are used to provide USSPT distances and LSI.

## Methods

### Design

A test-retest procedure was applied, with two testing sessions performed one week apart. All measurements were performed by one examiner, highly experienced with the testing procedure. The second session was performed under the same conditions as the first one, i.e. same procedure at the same time of day and with the same instructions for execution.

### Participants

*A priori* sample calculation estimated a sample size of 28 participants when considering a range of 0.30 for the confidence interval at 95 % confidence level of the intraclass correlation coefficient [13]. A convenient sample of 27 male athletes from several university sport teams and associations participated in this study, which was approved by the Ethics Committee (#2018-A03013-52). Inclusion criteria required being aged from 18 to 30 years old, practicing sport activity and being without upper limb and shoulder troubles at the time of the tests. Exclusion criteria were being injured in the upper limb during the six months preceding the study or having undergone surgery at the upper limbs.

### Procedure

**Fig. 1:**
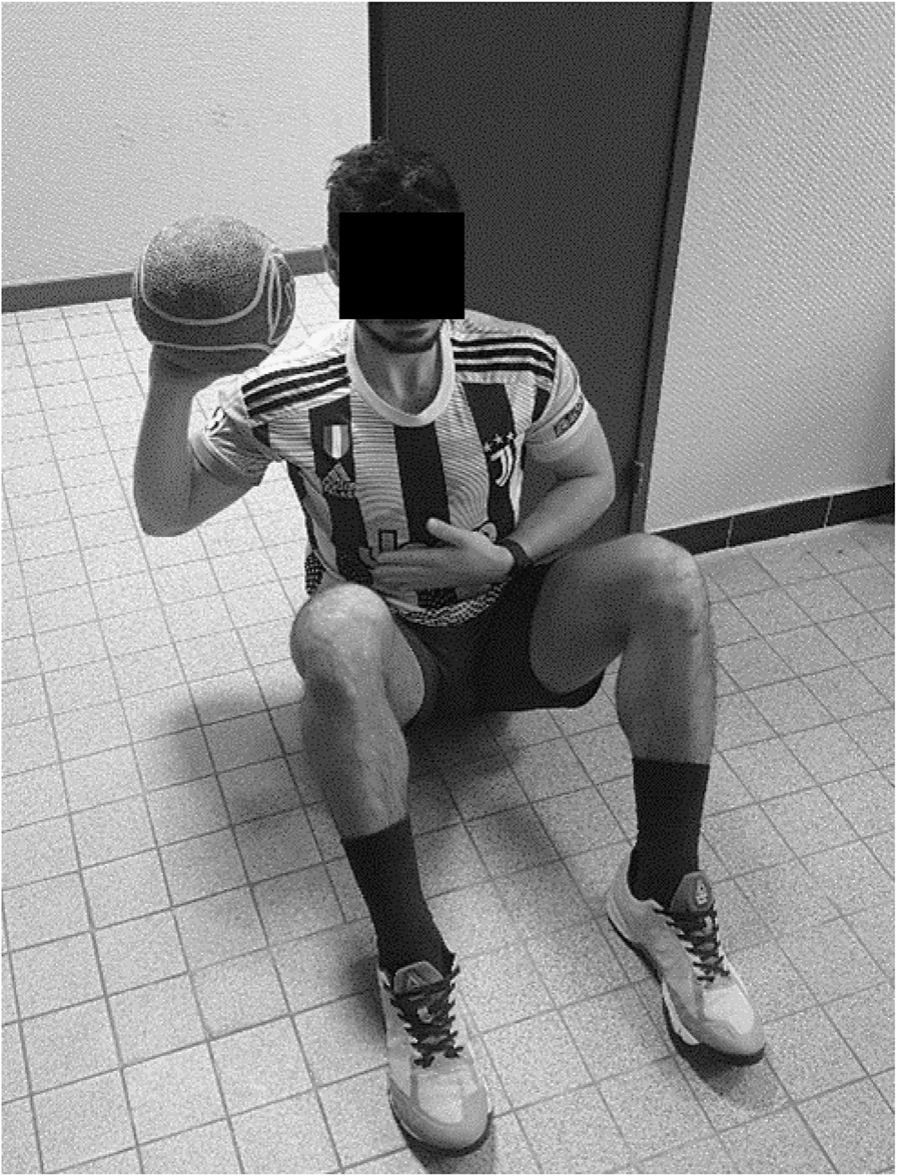
Starting position for the Unilateral Seated Shot-Put Test

At the beginning of each session and after a standardized warm-up [14], participants watched an instructional video describing the USSPT procedure. The participants sat on the floor, with knees flexed at 90° and feet flat, while half of his back and head kept contact with the wall. The participants were instructed to hold a 3-kg medicine ball [10, 12] at shoulder-height while flexing the elbow and then to push it as far as possible in the horizontal direction, with the opposite hand placed on the belly (Fig. 1). The right side was assessed first. Each testing session began by one submaximal and one maximal trials for familiarization during which the examiner gave additional instructions on the pushing direction if necessary. Then the participant performed three maximal trials for assessment. A 30-s recovery period was set between each trial. The medicine ball was coated with talcum powder to identify its impact on the ground. Each maximal trial was performed under vocal encouragement and supervised by an examiner to minimize the risk of any arm countermovement and ensure that the medicine ball was released along a horizontal direction. Any countermovement or parabolic trajectory cancelled the trial, and a new maximal trial was initiated until three trials were achieved in correct form. For each trial, the distance between the wall and the talcum mark (edge closest to the wall) was measured in centimeter [15]. After deducting the C7-middle finger length of the corresponding sides, the distances were scaled allometrically as proposed by Chmielewski et al. [8] (i.e. distance / body mass with the exponent 0.35).

### Statistical analysis

Based on recommendations outlined in the GRRAS [16], the intraclass correlation coefficient (3,k) (ICC) at a 95 % confidence level was used to assess the intra- and intersession reliability. ICC values higher than 0.70 indicated good reliability, values between 0.40 and 0.69, fair reliability, and values less than 0.40, poor reliability. The agreement was based on the standard error of measurement (SEM) at a 95 % confidence level, the minimal detectable change at a 95 % confidence level (MDC_95 %_), the coefficient of variation (CV) and Bland-Altman plots. Variability was acceptable when the CV value was lower than 12 % and unacceptable when the CV value was higher than 20 % [17]. The intrasession reliability was first examined for the normalized pushing distances between the first and second trials, between the second and third trials and between the three trials. Second, the intersession reliability was computed for normalized pushing distances independently for the first, second, third and best trials and means between the first and second trials, between the second and third trials and between the three trials. Third, among these computation methods, the one presenting the highest ICC values, and the lowest SEM, MDC_95 %_ and CV values simultaneously for both the dominant and non-dominant limbs was identified as USSPT pushing distances, and used to compute LSI, by dividing the pushing distance value of dominant limb by that of non-dominant limb. Finally, the intersession reliability and agreement of the LSI was assessed. The software SPSS 11.0 was used for all the statistical tests.

## Results

Twenty-two right-handed and five left-handed athletes (age: 22.5 ± 3.2 years; height: 1.77 ± 0.07 m; mass: 79.9 ± 9.1 kg; sport experience: 9.4 ± 5.4 years; weekly training: 8.56 ± 5.8 h) practicing various sport, such as rugby (*n* = 11), judo (*n* = 5), soccer (*n* = 3), strength training (*n* = 2), basketball (*n* = 2), climbing (*n* = 1), volleyball (*n* = 1), yoga (*n* = 1) and running (*n* = 1) participated in this study. On average, each participant performed seven trials in order to obtain six correct trials. Raw and normalized pushing distance values for each trial, the best trial and means between trials 1 and 2, trials 2 and 3 and the three trials for the dominant and non-dominant limbs are presented in Table 1.

**Table 1:** Mean ± standard deviation [minimal; maximal values], pushing raw and normalized distances, for each trial

Dominance	Trial	Session 1 (cm)	Session 2 (cm)
Raw			
Dominant	1	341 ± 63 [239;494]	339 ± 61 [229;478]
	2	346 ± 68 [226;495]	348 ± 69 [232;556]
	3	348 ± 75 [232;577]	346 ± 66 [236;566]
	Best trial	365 ± 73 [239;577]	358 ± 69 [236;566]
	Mean (1,2)^a^	343 ± 64 [232;494]	344 ± 63 [230;516]
	Mean (2,3)^b^	347 ± 69 [229;536]	347 ± 67 [234;561]
	Mean (1,2,3)^c^	345 ± 66 [232;522]	344 ± 64 [232;533]
Non-dominant	1	309 ± 64 [212;466]	319 ± 57 [189;416]
	2	322 ± 61 [189;446]	321 ± 59 [194;437]
	3	327 ± 73 [189;463]	319 ± 63 [224;456]
	Best trial	343 ± 65 [223;466]	337 ± 59 [224;456]
	Mean (1,2)	315 ± 60 [206;446]	320 ± 56 [192;422]
	Mean (2, 3)	324 ± 65 [189;454]	320 ± 58 [209;441]
	Mean (1,2,3)	319 ± 63 [200;446]	320 ± 57 [202;433]
Normalized			
Dominant	1	74 ± 13 [53;101]	73 ± 12 [51;97]
	2	75 ± 13 [50;105]	75 ± 14 [51;113]
	3	75 ± 15 [51;117]	75 ± 14 [52;115]
	Best trial	79 ± 15 [53;117]	77 ± 14 [52;115]
	Mean (1,2)	74 ± 13 [51;101]	74 ± 13 [51;105]
	Mean (2,3)	75 ± 14 [51;109]	75 ± 14 [52;114]
	Mean (1,2,3)	75 ± 13 [51;106]	74 ± 13 [51;108]
Non-dominant	1	67 ± 13 [46;95]	69 ± 12 [41;87]
	2	70 ± 13 [41;91]	69 ± 13 [42;94]
	3	71 ± 15 [41;94]	69 ± 13 [49;93]
	Best trial	74 ± 13 [49;95]	73 ± 12 [49;94]
	Mean (1,2)	68 ± 12 [45;91]	69 ± 12 [42;86]
	Mean (2,3)	70 ± 13 [41;92]	69 ± 12 [46;90]
	Mean (1,2,3)	69 ± 13 [44;91]	69 ± 12 [44;88]

^a^for the mean between the first and the second trial

^b^for the mean between the second and the third trial

^c^for the mean between the first, the second, and the third trial

The intrasession reliability and agreement of the normalized pushing distances (Table 2) were good between the first and second trials, the second and third trials and the three trials for each session for the dominant and non-dominant limbs.

**Table 2:** Intrasession reliability and agreement for normalized pushing distances, with [confidence interval at 95%]

	Set	ICC^d^	SEM (cm/kg^0.35^)^e^	MDC_95%_ (cm/kg^0.35^)^f^	CV (%)^g^
Session 1					
Dominant	1,2^a^	0.93 [0.84;0.97]	3 [2;5]	10	7.56
	2,3^b^	0.86 [0.68;0.94]	5 [4;8]	15	9.21
	1,2,3^c^	0.90 [0.78;0.96]	4 [3;6]	13	8.39
Non-dominant	1,2	0.86 [0.68;0.94]	4 [3;7]	14	10.09
	2,3	0.92 [0.81;0.96]	4 [3;6]	11	8.57
	1,2,3	0.90 [0.78;0.96]	4 [3;6]	12	9.33
Session 2					
Dominant	1,2	0.89 [0.73;0.95]	4 [3;6]	13	6.56
	2,3	0.98 [0.95;0.99]	2 [1;3]	6	6.34
	1,2,3	0.94 [0.85;0.98]	3 [2;5]	10	6.45
Non-Dominant	1,2	0.81 [0.57;0.91]	5 [4;8]	15	8.70
	2,3	0.74 [0.43;0.88]	6 [4;9]	18	11.0
	1,2,3	0.78 [0.55;0.91]	6 [4;8]	17	9.87

^a^between the first and second trials

^b^between the second and third trials

^c^between the three trials

^d^for intraclass coefficient of correlation

^e^for standard error of measurement

^f^for minimal detectable change at a 95% confidence level

^g^for the coefficient of variation

The intersession reliability and agreement of the normalized pushing distances (Table 3) were good for the first, second, third and best trials for the dominant and non-dominant limbs. Nevertheless, when considering both sides, the highest reliability and the lowest agreement values were found for the mean of the three trials (Mean (1,2,3) in Table 3).

**Table 3:** Intersession reliability and agreement for normalized pushing distances, with [confidence interval at 95%]

	ICC^d^	SEM (cm/kg^0.35^)^e^	MDC_95%_ (cm/kg^0.35^)^f^	CV (%)^g^
Dominant				
1	0.85 [0.66;0.93]	5 [3;7]	13	8.63
2	0.80 [0.55;0.90]	6 [4;9]	17	10.0
3	0.94 [0.85;0.97]	3 [2;5]	10	7.62
Best trial	0.95 [0.89;0.98]	3 [2;5]	9	7.59
Mean (1,2)^a^	0.88 [0.71;0.94]	4 [3;7]	13	7.30
Mean (2,3)^b^	0.91 [0.78;0.96]	4 [3;6]	12	6.98
**Mean (1,2,3)**^**c**^	**0.92 [0.81;0.96]**	**3 [2;5]**	**10**	**6.23**
Non-dominant				
1	0.79 [0.52;0.90]	5 [4;8]	16	11.5
2	0.86 [0.67;0.93]	4 [3;7]	13	8.81
3	0.83 [0.60;0.92]	5 [4;8]	16	10.9
Best trial	0.90 [0.78;0.95]	4 [3;6]	11	8.33
Mean (1,2)	0.90 [0.76;0.95]	3 [2;5]	10	7.26
Mean (2,3)	0.90 [0.77;0.95]	4 [3;6]	11	7.10
**Mean (1,2,3)**	**0.93 [0.82;0.97]**	**3 [2;5]**	**9**	**6.06**

^a^for the mean between the first and the second trial

^b^for the mean between the second and the third trial

^c^for the mean between the first, the second, and the third trial

^d^for intraclass coefficient of correlation

^e^for standard error of measurement

^f^for minimal detectable change at a 95% confidence level

^g^for the coefficient of variation

**Fig. 2:**
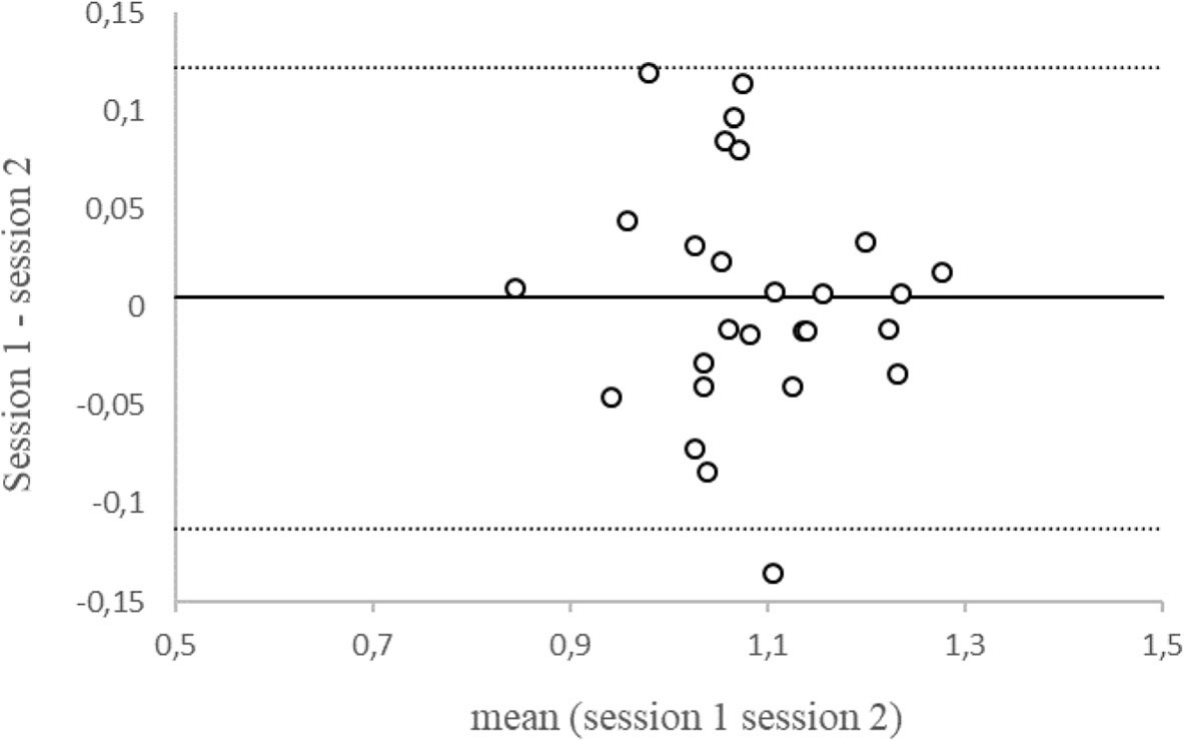
Bland-Altman plot between the first and second sessions for LSI

The mean normalized distances of the three trials were used to compute the LSI by dividing the value of the dominant limb by that of the non-dominant limb. The mean LSI was 1.09 ± 0.10 for the first session and 1.08 ± 0.10 for the second session. The intersession reliability and agreement of the LSI were good (ICC = 0.82 [CI_95 %_: 0.59; 0.91]; SEM = 0.045 [CI_95 %_: 0.03; 0.06]; MDC_95 %_ = 0.124; CV = 5.02 %). The Bland-Altman plot illustrates that measurements for 26 of 27 individuals (96.2 %) were within the limits of agreement [-0.11; 0.12] (Fig. 2). The bias was 0.005, indicating a slightly higher score for the first session than the second session.

## Discussion

This study aimed at assessing the reliability and agreement of USSPT outcome measures based on one, two or three trials per side when pushing the overweight ball in a horizontal direction. The main findings were that best reliability and agreement criteria values for USSPT distances and LSI were found when averaging the distances of three trials for both the dominant and non-dominant limbs.

When performed unilaterally, the medicine ball shot-put test provides a pushing distance for each upper extremity, allowing the bilateral comparison of shoulder and upper limb function. Our mean raw distances ranged from 226 to 577 cm and from 189 to 466 cm for dominant and non-dominant limbs, respectively, confirming that pushing distances for the dominant side are higher than for non-dominant side [7–9]. These values were slightly lower than those reported for healthy athletes [8] and similar to those reported for athletes presenting chronic shoulder pain [10]. Such discrepancies may be explained by differences in pushing patterns, i.e. horizontal direction vs. standard shot-put technique [8, 10], and sport specificity, i.e. multisport vs. overhead sport athletes [8]. Nevertheless, when comparing the pushing distances of the dominant and non-dominant sides, we found similar limb symmetry indices than those described by Chmielewski et al. [8]. In consequence, when comparing studies, the direction of the pushing should be considered when the performance of each limb is assessed independently. By contrast, controlling the pushing direction seems to be less relevant when the bilateral symmetry is of interest.

The implementation of a physical performance test into a battery demands that the main outcome measures of such a test are statistically valid [6] while ensuring high efficiency and low costs. Requiring few materials, the USSPT procedure has already demonstrated good reliability in healthy and symptomatic athletes when performance was computed by averaging the distance of three trials [10, 11]. In a clinical context, using the optimal number of trials may help to save time and optimize the testing procedure. Either for single or repeated sessions, reducing the number of trials is not advised since the best reliability and agreement criteria values, when both the sides considered, were obtained for USSPT outcome measures based on three trials. Based on SEM values, significant changes in USSPT pushing distances may be considered for variations higher than 13–26 cm for the dominant and non-dominant limbs, respectively, and 4 % for USSPT LSI. Our findings indicate that when the overweight ball is pushed in horizontal direction, the averaged USSPT pushing distance from three trials and derived LSI are reliable outcome measures, which may be used routinely by coaches and clinicians for upper extremity functional assessment.

Some limitations should be considered in this study. First, non-negligible MDC_95 %_ values found in our series may be partly explained by heterogeneity in the sport practices of our athletes, and further studies are required to better understand performance variability according to sport specificity or practice level. Second, only three maximal trials were assessed per limb, based on the usual USSPT procedure [7, 8, 11]. Implementing additional trials may improve reliability and agreement criteria; however, the implementation may be too time-consuming when the USSPT is included in a battery of tests, limiting its use by sport and clinical practitioners for the comprehensive assessment of upper extremity function. Third, as only healthy male athletes were included in this study, reliability and agreement of the USSPT distances and LSI cannot be generalized for population of other sex, with history of upper extremity injuries or for patient during rehabilitation. Nevertheless, the findings of this study may be useful for strength and conditioning coaches or clinicians to assess upper extremity power function with easy-to-use physical performance test and reliable outcomes measures.

## Conclusions

The findings of this study indicate that when the overweight ball is pushed in horizontal direction to perform USSPT, averaging the pushing distances of three trials is advised since it provides the most reliable outcome measures both for the dominant and non-dominant limb. For bilateral balance assessment, the USSPT LSI demonstrates good intersession reliability and agreement.

## Data Availability

The datasets used and/or analysed in this study are available from the corresponding author on request.

## Abbreviations

USSPT: Unilateral Seated Shot-Put Test
LSI: Limb symmetry index
ICC: Intraclass correlation coefficient
SEM: Standard error of measurement
MDC_95 %_: Minimal detectable change at a 95 % confidence level
CV: Coefficient of variation
CI_95 %_: 95 % confidence interval
